# Astrovirus MLB1 Is Not Associated with Diarrhea in a Cohort of Indian Children

**DOI:** 10.1371/journal.pone.0028647

**Published:** 2011-12-09

**Authors:** Lori R. Holtz, Irma K. Bauer, Priya Rajendran, Gagandeep Kang, David Wang

**Affiliations:** 1 Washington University School of Medicine, Department of Pediatrics, St. Louis, Missouri, United States of America; 2 Washington University School of Medicine, Departments of Molecular Microbiology and Pathology and Immunology, St. Louis, Missouri, United States of America; 3 Christian Medical College, Department of Gastrointestinal Sciences, Vellore, India; University of Hong Kong, Hong Kong

## Abstract

Astroviruses are a known cause of human diarrhea. Recently the highly divergent astrovirus MLB1 (MLB1) was identified in a stool sample from a patient with diarrhea. It has subsequently been detected in stool from individuals with and without diarrhea. To determine whether MLB1 is associated with diarrhea, we conducted a case control study of MLB1. In parallel, the prevalence of the classic human astroviruses (HAstVs) was also determined in the same case control cohort. 400 cases and 400 paired controls from a longitudinal birth cohort in Vellore, India were analyzed by RT-PCR. While HAstVs were associated with diarrhea (p = 0.029) in this cohort, MLB1 was not; 14 of the controls and 4 cases were positive for MLB1. Furthermore, MLB1 viral load did not differ significantly between the cases and controls. The role of MLB1 in human health still remains unknown and future studies are needed.

## Introduction

The first astrovirus infecting humans was described in 1975 [1]. Since then, a total of 8 serotypes closely related to this original astrovirus (“classic human astroviruses” (HAstVs)) have been identified, all of which are believed to cause diarrhea. Diarrhea symptoms typically last 2–4 days following a 3–4 day incubation period [2]. These infections most commonly affect children, the elderly, and the immunocompromised [3]. HAstVs account for up to ∼10% of sporadic cases of non-bacterial diarrhea in children [4], [5], [6], [7]. Since 2008, five highly divergent astroviruses have been discovered in human diarrhea specimens: MLB1 [8], astrovirus MLB2 (MLB2) [9], astrovirus VA1 (VA1) [10], astrovirus VA2 (VA2) [9], and astrovirus VA3 (VA3) [9]. Of these viruses, MLB1 has been detected at the highest frequency. MLB1 was first identified in the stool of child with unexplained diarrhea. Subsequently, it has been found in stools collected from around the world [9], [11], [12], [13], [14], [15] from patients with and without diarrhea.

The finding of the novel astrovirus MLB1 in stool specimens from patients with diarrhea raises the question of whether or not, like all other human astroviruses, it also causes diarrhea. Historically, proof of causality for diarrheagenic viruses relied upon ingestion of fecal filtrates by human volunteers [2], [16], [17], [18]. Such studies are, however, no longer feasible for novel viruses of unknown pathogenicity. Thus, assigning pathogenicity to a newly identified virus requires indirect approaches such as infection of surrogate animal models or epidemiologic analyses of human populations. Here we describe a case control study aimed at determining whether or not MLB1 is associated with diarrhea.

## Materials and Methods

### Description of Samples

The institutional review boards of Christian Medical College, Vellore, India and Washington University School of Medicine, St. Louis, USA approved this study. 400 case and 400 control stool samples were selected from a previously described longitudinal birth cohort in Vellore, India [19], [20], [21]. Children were followed for 3 years with twice-weekly home visits and collection of stool every two weeks and during every diarrheal episode. 373 children completed the three year study. A total of 1955 diarrhea and ∼27,000 control stools were collected from 2002–2006. The severity of each diarrheal episode was recorded using the 20 point Vesikari scale developed for rotaviral gastroenteritis, which includes number and duration of diarrhea and vomiting episodes, presence of fever and dehydration and classifies gastroenteritis as mild, moderate, severe and very severe [22]. 400 stool samples from acute diarrhea episodes that were negative for rotavirus by enzyme immunoassay and PCR, for norovirus by PCR, for bacterial pathogens (*Vibrio cholerae*, enteropathogenic *Escherichia coli*, *Salmonella*, *Shigella*, *Aeromonas* and *Plesiomonas*) by culture, biochemical reactions and serogrouping where appropriate and for parasites by routine saline and iodine preparations and modified acid fast stain were chosen as cases [23]. To obtain paired control samples, an asymptomatic surveillance stool sample collected at least 6 weeks prior to the acute diarrhea sample was selected from the same child. The 400 paired samples were collected from 249 children.

### RT-PCR

200 µL of a ∼20% fecal suspension were extracted using the Boom method [24] and the extracted total nucleic acid was eluted into 40 µL of water. As previously described, a two stage screening strategy was used to detect astroviruses [9]. In brief, astrovirus consensus primers SF0073 (5′-GATTGGACTCGATTTGATGG-3′) and SF0076 (5′-CTGGCTTAACCCACATTCC-3′), designed to the RNA polymerase (ORF 1b) were used to screen all samples in the first stage. Samples that were positive in the first phase of screening were then subjected to additional RT-PCR screenings with primers specific for classic human astroviruses [Mon269 (5′-CAACTCAGGAAACAGGGTGT-3′) and Mon270 (5′-TCAGATGCATTGTCATTGGT-3′)] [25] and primers specific for MLB1 [SF0053 (5′-CTGTAGCTCGTGTTAGTCTTAACA-3′) and SF0061 (5′-GTTCATTGGCACCATCAGAAC-3′)], both of which target the capsid region (ORF 2). PCR amplicons were cloned into pCR4 (Invitrogen) and sequenced using standard Sanger sequencing technology.

### Phylogenetic Analysis

Sequences of the capsid region amplicons from HAstV and MLB1 positive samples were aligned using ClustalX1.83. PAUP was then used to generate maximum parsimony trees with 1,000 bootstrap replicates.

### qRT-PCR

MLB1 consensus primers ( LG0189 5′- AAGTGTGCATATGTTGGGACC -3′ and LG0190 5′- CTACACCTCTCCAATTCATG -′3) targeting a 131 nt segment of the ORF1a region of the MLB1 genome were designed by alignment of the 4 MLB1 sequences present in GenBank (NC_011400.1, FJ402983.1, HM450380.1, and HM989952.1) containing the region of interest as of 12/23/2010; these primers were used in a SYBR green qRT-PCR assay. QRT-PCR was performed using qScript One-Step kit (Quanta) as follows: 50°C for 10 min, 95°C for 5 min, 45 cycles of 95°C for 10 sec and 60°C for 30 sec followed by a melt curve. To establish a standard curve for this assay, in-vitro transcribed RNA was generated from a plasmid containing nt 1 to 846 of MLB1 (GenBank NC_011400.1) using MAXIscript (Ambion) per manufacturer's protocol. Serial dilutions of this in-vitro transcribed RNA from 5×10^6^ copies to 5 copies were used for the standard curve.

## Results

### RT-PCR case control study

For HAstVs, 14 of the 400 cases were positive and 4 of the 400 controls were positive. By contrast, for MLB1 only four of the cases were positive while 14 of the controls were positive. To take into account the fact that some participants were sampled more than once, logistic regression models were fit using generalized estimating equations. HAstVs were more likely to be present in the diarrheal samples than in the asymptomatic samples (OR 3.59, 95% CI 1.14–11.31, p = 0.029), consistent with results of previous studies [26]. By contrast MLB1 was less likely to be present in the diarrheal samples than in the asymptomatic samples (OR 0.28, 95% CI 0.09–0.89, p = 0.033).

All RT-PCR positive samples were cloned and sequenced (GenBank JN871233–JN871268). Amplicons from the capsid region were chosen for sequencing, as this is the least conserved region of the genome and might reveal the most divergence. The MLB1 capsid amplicons shared 96–99% nucleotide identity to each other while the HAstV capsid amplicons shared 77–100% nucleotide identity to each other. Phylogenetic analysis of the HAstV and MLB1 amplicons obtained in the capsid regions showed no clustering of the cases vs controls (data not shown). Five samples (4 controls and 1 case) were positive for both HAstV and MLB1.

Of the 249 children studied 107 were sampled at multiple time points. One subject had two diarrhea samples positive for HAstVs separated by 14 months. The intervening control stool, collected 12 months after the 1^st^ diarrhea sample and 7 weeks before the 2^nd^ diarrhea sample, from this subject was negative for HAstVs. The capsid-derived amplicons from these two diarrhea samples shared 78% identity, suggesting that this subject was infected by two different HAstV serotypes at the two time points. Two subjects had two asymptomatic stools positive for MLB1 with an intervening diarrhea sample that was negative for MLB1. For one subject these asymptomatic samples were separated by 18 months. The capsid-derived amplicons from these two samples shared 98% identity. For the other subject, these asymptomatic samples were separated by 8 months and shared 99% identity. One interpretation of this observation is that reinfection by MLB1 is possible.

### qRT-PCR of positive samples

Samples positive for MLB1 by RT-PCR were then subjected to qRT-PCR to establish if viral load differed between cases and controls as has been described for norovirus [27]. There was no significant difference in the RNA copy number/ml of fecal suspension between cases and controls. Specifically, for the 4 cases the average RNA copy number/ml fecal suspension was 7×10^3^ and for the 14 positive control samples the average copy number/ml of fecal suspension was 4×10^4^. Using the Mann-Whitney test, there was not a significant difference between cases and controls (p = 0.51).

## Discussion

In this study, we performed a case control study of MLB1 and HAstVs. We demonstrated in this cohort that HAstVs are associated with diarrhea. By contrast, MLB1 was not associated with diarrhea. These results suggest that MLB1 may not play an etiologic role in human diarrhea. However, a single case control study is not sufficient to definitively answer this question as evidenced by the fact that there are examples from the literature of bona fide diarrhea pathogens for which case control studies did not yield positive associations. For example, a case control study of *Campylobacter* found that it was present in 22% of cases vs. 25% of controls [28]. Furthermore, *Giardia lamblia* was found more commonly in controls than in cases in two studies [28], [29]. A case control study from Vietnam found diarrheagenic *E.coli* in 23% of cases and 23% of controls [30]. These studies highlight the challenges of determining association of a microbe with a given disease strictly by a case control study. The role of MLB1 in human health remains uncertain. It remains possible that MLB1 is an agent of diarrhea. One formal possibility is that it is present in stool simply as a result of dietary ingestion and it has no pathogenic role. Another possibility is that MLB1's pathogenic effects are outside of the enteric system and that its detection in stool simply reflects its mode of transmission, much like poliovirus. In support of this possibility, we have recently described a febrile child with MLB2 viremia, demonstrating that astroviruses can access the circulatory system and thus could have broader tropism outside the GI tract [31]. In addition, another astrovirus was recently found in the brain tissue of an immunocompromised patient with encephalitis [32]. Further studies are still needed to establish the role of MLB1 in human health and disease.
